# Mix and (mis-)match – The mechanosensing machinery in the changing environment of the developing, healthy adult and diseased heart^☆^

**DOI:** 10.1016/j.bbamcr.2019.01.017

**Published:** 2020-03

**Authors:** Matthew Ward, Thomas Iskratsch

**Affiliations:** Division of Bioengineering, School of Engineering and Materials Science & Institute for Bioengineering, Queen Mary University of London, United Kingdom

**Keywords:** CM, Cardiomyocytes, HCM, hypertrophic cardiomyopathy, DCM, Dilated cardiomyopathy, ICM, Idiopathic cardiomyopathy, MYH, Myosin Heavy Chain, TNNT, Troponin T, TNNI, Troponin I, AFM, atomic force microscope, MRE, magnetic resonance elastography, SWE, ultrasound cardiac shear-wave elastography, LV, Left ventricle, LOX, Lysyl oxidase, LOXL, Lysyl oxidase like protein, LH, Lysyl hydroxylase, Lys, Lysin, LCCs, Lys^ald^-derived collagen crosslinks, HLCCs, Hyl^ald^-derived collagen crosslinks, PKA, Protein Kinase A, PKC, Protein Kinase C, VASH1, vasohibin-1, SVBP, small vasohibin binding protein, TCP, tubulin carboxypeptidase, TTL, tubulin tyrosine ligase, MRTF, myocardin-related transcription factor, GAP, GTPase activating protein, GEF, Guanine nucleotide exchange factor

## Abstract

The composition and the stiffness of cardiac microenvironment change during development and/or in heart disease. Cardiomyocytes (CMs) and their progenitors sense these changes, which decides over the cell fate and can trigger CM (progenitor) proliferation, differentiation, de-differentiation or death. The field of mechanobiology has seen a constant increase in output that also includes a wealth of new studies specific to cardiac or cardiomyocyte mechanosensing. As a result, mechanosensing and transduction in the heart is increasingly being recognised as a main driver of regulating the heart formation and function. Recent work has for instance focused on measuring the molecular, physical and mechanical changes of the cellular environment - as well as intracellular contributors to the passive stiffness of the heart. On the other hand, a variety of new studies shed light into the molecular machinery that allow the cardiomyocytes to sense these properties. Here we want to discuss the recent work on this topic, but also specifically focus on how the different components are regulated at various stages during development, in health or disease in order to highlight changes that might contribute to disease progression and heart failure.

## Highlights

• The stiffness of the heart changes during development or in disease.
• These changes influence cardiomyocyte behaviour and function.
• Various intra- and extracellular elements contribute to the stiffness.
• The molecules and pathways that are sensing the stiffness are changing as well.

## 1 Introduction

The heart is the first functional organ in the body. Once the organism grows beyond a size where diffusion is sufficient to distribute oxygen and nutrients, it relies on the ability of the heart to efficiently pump blood around the body. As the organism grows, the heart has to adapt to the changing demands and generate the increasing blood pressures, necessary for the blood circulation.

For this, cardiomyocytes have to contract in a controlled and concerted fashion. For communication the cardiomyocytes are coupled chemically, electrically and mechanically. The mechanical coupling is achieved through direct cell-cell coupling at the intercalated disc, as well as the extracellular matrix to which the cardiomyocytes are attached laterally at the so-called costameres through integrins and integrin associated proteins, as well as the dystrophin-glycoprotein complex.

Recent data increasingly point to a major role for mechanical forces for a variety of cellular processes in many cell types, including cardiomyocytes [1]. While there have been several excellent recent reviews discussing how mechanosensing and -transduction influence cell phenotypes, migration, differentiation or health, the forces cardiomyocytes experience are unique, owing to the regular cyclic contraction and haemodynamic forces from filling of the heart with blood. Moreover, cardiomyocytes differ from other cell types in terms of cytoskeletal organisation, proteome and metabolism. This suggests a unique mechanotransduction system, which indeed has been indicated by a range of recent studies.

We will focus here on the recent advances and current state of research of mechanical sensing in cardiomyocytes and the impact on health and disease. Importantly, both maturity of the cardiomyocytes and the cellular environment change during the development and in cardiac disease, transforming the force landscape that the cardiomyocytes are exposed to. These changes include also ventricular pressure, e.g. after birth, when the loss of blood flow through the placenta leads to a rise in vascular resistance and increase in left ventricular pressure. Further, the opening of the lungs results in a drop in pulmonary resistance and decreasing right ventricular pressure [[2], [3], [4], [5]]. This has also consequences for cardiomyocyte growth and hypertrophy, leading to left ventricular wall thickening and right ventricular wall thinning. At the same time altered cardiac signalling pathways (e.g. due to inflammation) cause a change in the expression pattern of molecules that are involved in mechanosensing and transduction.

In this review we will first discuss the changing environment and forces in the heart, before we look at the mechanosensing machinery and how changes in its components can contribute to the disease progression.

## 2 Changing mechanical properties in the heart

Mechanotransduction depends on the conversion of mechanical forces into chemical signals [1]. Mechanosensitive proteins sit at cell-cell and especially cell-matrix adhesions, where (active and passive) forces onto the adhesions lead to deformation (e.g. opening of cryptic binding sites), binding, or unbinding of mechanosensors. These events depend on one hand on the mechanical properties of the extracellular matrix, but also on the binding affinities and force dependency of the receptor-ligand interaction as well as the composition and mechanical properties of the adhesion complex [6].

Cardiomyocytes sense both active contractile forces and passive stiffness (i.e. the stiffness that is independent of muscle activity) at single adhesions, with a major influence of the passive stiffness as modulator of the mechanical signalling [7]. Active force production changes during development or in disease, because of the altered expression of motor proteins and their regulators as well as the changing load and its effect on the cardiac output [5,8,9]. But inadequate force production or transmission can also lead to cardiac remodelling (HCM or DCM). E.g. mutations in *MYH7* (Ser532Pro), *TNNT2* (ΔLys210) and *TNNI3* (Ala2Val) are linked to reduced contractile forces, DCM and heart failure [10]. While in case of HCM only a small subset of patients will develop heart failure, different mutations in the same set of genes listed above have been linked to especially fast clinical deterioration because of systolic dysfunction and reduced ejection fraction [10]. However, nearly half of subjects with heart failure suffer from diastolic dysfunction, which is mainly determined by altered passive stiffness [11]. These patients are typically diagnosed with heart failure with preserved ejection fraction (HFpEF) (which was in the past also referred to as diastolic heart failure), although diastolic dysfunction can also exist in heart failure with reduced ejection fraction (HFrEF). The transition from the compensated stage to diastolic heart failure is especially linked with progressive myocardial stiffening [[12], [13], [14]].

Several studies have so far attempted to measure the changes to the heart stiffness either during development or in disease [[15], [16], [17], [18], [19], [20], [21], [22], [23], [24]]. These measurements vary substantially, depending on the experimental setup and the model system, but also within single studies (see Table 1). E.g. micropipette measurements indicated a young's modulus (a measure of a material to resist elastic deformation when under lengthwise tension or compression) of native adult rat heart tissue that ranged between 11.9 and 46.2 kPa (mean 25.6 ± 15.9 kPa) [16]. AFM measurements on the other hand reported 18 ± 2 kPa for healthy left ventricles (compared to 55 ± 15 kPa in a model for myocardial infarction) [17]. While some of these studies are limited by an extremely low n-number, they do generally show that the stiffness of the samples is not homogeneous within a sample and might also vary between individuals or species as much as between different areas of the heart. Over the last few years, this has been indeed confirmed by in vivo measurements using cardiac magnetic resonance elastography (MRE) and ultrasound cardiac shear-wave elastography (SWE). Recent advances of these non-invasive techniques enable now direct comparison between diastolic and systolic values and measurements in human individuals. Moreover, detailed stiffness maps can be generated, which indicate significant variations of the stiffness within different regions of the heart [18,24].

**Table 1:** Studies investigating the stiffness of the heart. For SWE and MRE, the printed Young's modulus represents the calculated values, using the shear modulus from the original studies and a Poisson's ratio of 0.4 [[15], [16], [17], [18], [19], [20], [21], [22]].

Method	Species	Chamber	Condition	Shear modulus	Young's modulus	N-numbers	Ref
Micropipette	Chick	heart tube	Embryonic (E4)		~ 1 kPa	4	[15]
		heart tube	Embryonic (E14)		~ 10 kPa	1	
Micropipette	Rat	LV	Neonatal		6.8 ± 2.8 kPa	1	[16]
			Adult - healthy		25.6 ± 15.9 kPa	1	
AFM	Rat	LV	Adult - healthy		18 ± 2 kPa	2	[17]
			Adult - MI		55 ± 15 kPa	2	
Shear Wave	Sheep	Atrium	Adult - healthy	Diastole: 0.5 ± 0.1 kPa Systole: 6.0 ± 0.3 kPa	Diastole: 1.4 ± 0.3 kPa Systole: 16.8 ± 0.8 kPa	1	[18]
		RV		Diastole: 1.3 ± 0.3 kPa Systole:13.5 ± 9.1 kPa	Diastole: 3.6 ± 0.8 kPa Systole: 37.8 ± 25.5 kPa		
		LV		Diastole: 1.3 ± 0.2 kPa Systole:12.5 ± 5.1 kPa	Diastole: 3.6 ± 0.6 kPa Systole: 35.0 ± 14.2 kPa		
Magnetic Resonance Elastography	Pig	LV	Healthy	Diastole: 6.0 ± 1.8 kPa Systole: 9.3 ± 1.9 kPa	Diastole: 16.9 ± 5.0 kPa Systole: 26.2 ± 5.3 kPa	6	[19]
	Pig	LV	Juvenile – before surgery	Diastole: 3.9 ± 0.4 kPa Systole: 5.0 ± 0.7 kPa	Diastole: 10.8 ± 1.1 kPa Systole: 13.9 ± 2 kPa	21	[20]
			Juvenile – MI (21d)	Diastole: 5.5 ± 0.7 kPa Systole: 6.3 ± 1.0 kPa	Diastole: 15.3 ± 2 kPa Systole: 17.8 ± 2.8 kPa		
	Human	LV	Adult - healthy	Systole: 5.64 ± 1 kPa	Systole: 15.8 ± 2.8 kPa	18 (13 Female and 5 Male; Mean age: 31 yrs.; range: 21–71 yrs)	[21]
			Adult, Obstractive HCM	Systole: 14.5 ± 2.2 kPa	Systole: 40.6 ± 6.2 kPa	2 Female; Ages: 26 and 43 yrs)	
	Human	LV	Adult - healthy	early systolic phase: mean: 8.2 kPa; range: 7.2–11.8 kPa	early systolic phase: mean: 23.0 kPa; range: 20.2–33.0 kPa	11 (1 Female and 10 Male; Median age: 57; range: 52–84 yrs)	[22]
			Adult cardiac amyloid patients	early systolic phase: mean: 11.4 kPa: range: 9.2–15.7 kPa	early systolic phase: mean: 32.0 kPa; range 25.8–44.0 kPa	16 (3 Female and 13 Male; Median age 66.5; range: 50–85 yrs)	

On the other hand, these methods rely on computational models to calculate stiffness values and perhaps because of different setups and analytic approaches, the results vary again between different studies. Because both methods measure the shear modulus, Young's moduli were here calculated from the published shear moduli, assuming a Poisson's ratio (describes how much a material expands in the transverse direction after axial compression, i.e. the negative of the ratio between transverse and axial strain) of 0.4 for the myocardium [23] to allow comparison with micropipette and AFM measurements. Using SWE, diastolic Young's moduli of approx. 3.6 kPa were detected in sheep left ventricles (LV), compared to 35kPa during systole [18]. In contrast, diastolic MRE values were around 17 kPa in pig LVs, compared to ~26 kPa during systole [19]. Similar values were also found in one study investigating human LV stiffness [22], although somewhat lower stiffnesses were measured in another pig study as well as a second study using human LVs [20,21]. Noteworthy, apart from differences in the experimental setup as well as the image processing and analysis, sex and age spread were significantly different between the human studies, limiting their comparability.

The studies are however all consistent in the finding that stiffness goes up during development to a diastolic adult LV stiffness between 10 and 20 kPa (Table 1). Considering that the systolic values were around 50% higher compared to end-diastole, when measured by MRE, measurements from the human hearts also fall within this range. Similarly, all studies find that the stiffness is increased in diseased hearts.

## 3 Contributing elements to the cardiac stiffness

Passive stiffness is a key factor in the heart. It determines the filling and stroke volume, as well as the shortening velocity in the activated myocardium [25,26]. Also, as described above, cells in the myocardium, including the cardiomyocytes can sense the changing stiffness of the heart, thereby influencing their phenotype and behaviour. Therefore, the next section we will look at the main contributors towards the passive stiffness, including the extracellular matrix surrounding the cardiomyocytes, as well as intracellular elements, such as the giant sarcomeric protein titin or the microtubule network.

### 3.1 Extracellular stiffness - ECM and basal lamina composition

The cardiac extracellular environment is an intricate web of proteoglycans, glycosaminoglycans and glycoproteins, such collagens, fibronectin or laminin [27,28]. The fibrillar collagens I, III and V in the extracellular matrix (ECM) are connected through collagen VI, fibronectin and other molecules to the network forming collagen IV in the basal lamina, into which laminin, other glycoproteins and proteoglycans are incorporated. The composition of the extracellular matrix shows spatial and temporal changes during the cardiac development that include all the above molecules as well as matrix proteases. Together these changes contribute to the increase of the cardiac stiffness from the embryonic to the adult heart and further in diseased, fibrotic myocardium (see Section 2 and Table 1). The fibrillar collagens I,III,V and the network forming collagen IV are all expressed during development and their loss is lethal at embryonic stages (collagen I, IV, V) or in adulthood (type III) [27,29]. Fibronectin levels are high during embryonic development but decrease after birth, while laminin shows the opposite trend [30]. At the same time there is also a shift in the expression of the different laminin α-chain isoforms, from predominantly laminin α2 (which binds to α-dystroglycan in the dystrophin glycoprotein complex and α7β1 integrins) to predominantly laminin α5 (which has a much stronger affinity towards α7β1 integrins but doesn't bind to α-dystroglycan) [31,32]. However, especially the decrease in the ratio of Type I/Type III from neonatal to adult stages [33] and the increase in the crosslinking of the collagen [34] is associated with changes to the mechanical properties.

**Fig. 1:**
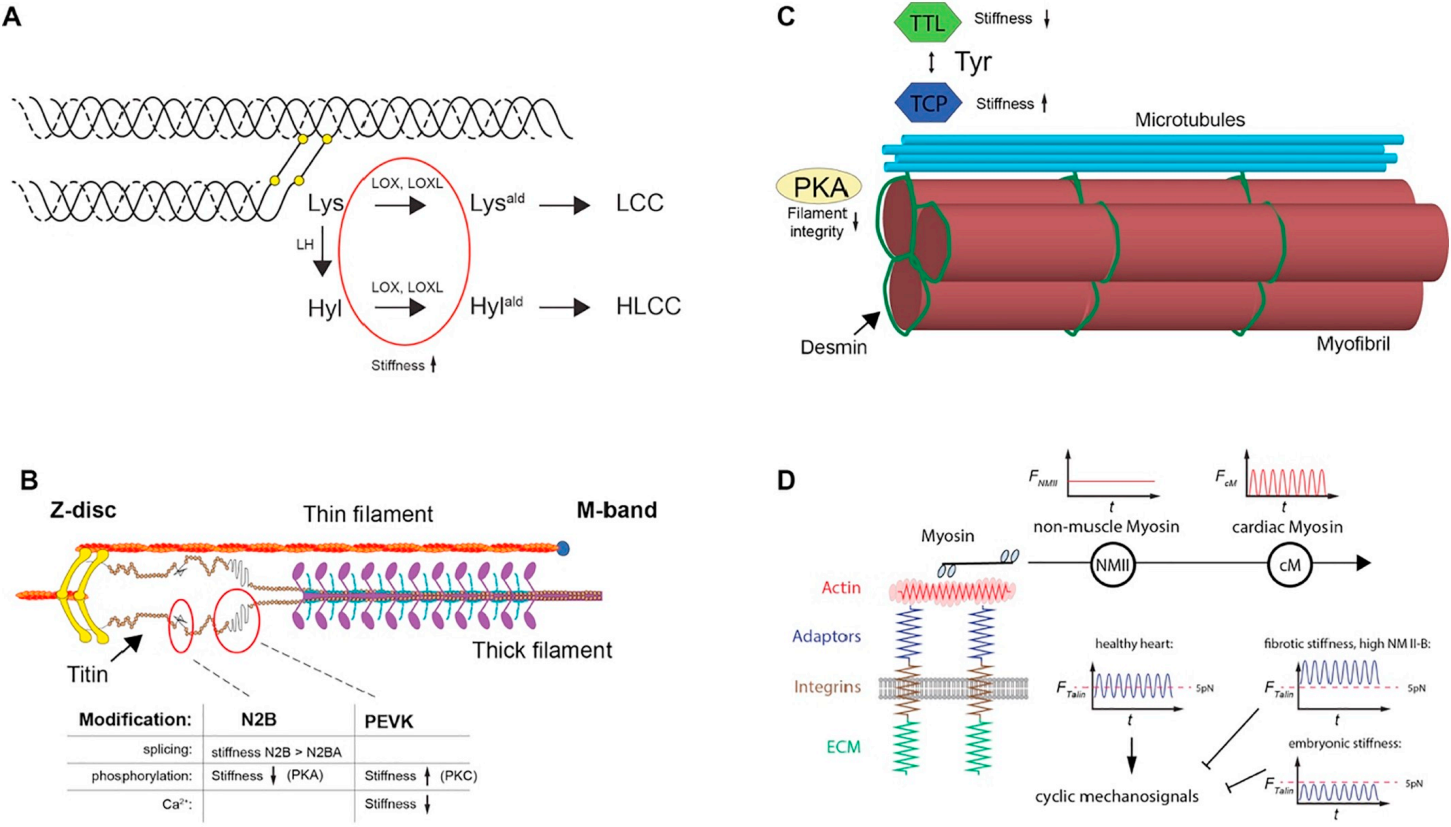
Main contributors to the passive stiffness in the heart. A) Collagen is crosslinked through lysyl oxidases (LOX, LOXL), increasing its stiffness. Additionally, the stability of the crosslinks is determined through activity of lysyl hydroxylases (LH). B) The stiffness of the elastic N2B and the PEVK domains of titin can be changed through splicing, phosphorylation or calcium binding. C) Detyrosination (through unknown tubulin carboxypeptidases) of microtubules leads to crosslinking with desmin and high-resistance buckling as opposed to low resistance sliding behavior. Detyrosination can be reversed by tubulin tyrosine ligase (TTL) activity. D) Non-muscle myosin can increase the basal tension that is sensed by cardiomyocyte integrin adhesions, thereby changing the mechanical signalling through adaptor proteins, such as talin (e.g. cyclic vs continuous stretching of talin) – figure adapted from Pandey et al., 2018 [7].

Therefore, unsurprisingly, collagen crosslinking is associated with systolic and especially diastolic dysfunction. The soluble pro-collagen I and III are secreted by myofibroblasts and then further crosslinked and assembled into fibres. There are two major groups of crosslinks: those initiated by the enzyme LOX and those derived from nonenzymatically glycated lysine (Lys) and hydroxylysine (Hyl) residues [35]. Additionally, the type of crosslinks that can be formed are determined by another class of enzymes the lysyl hydroxylases (LH), which hydroxylate lysine residues (Fig. 1A). LOX can oxidatively deaminate lysine residues that then form relatively unstable Lys^ald^-derived collagen crosslinks (LCCs). Alternatively, it can also modify Hyl which then can form the stable Hyl^ald^-derived collagen crosslinks (HLCCs). Generally, HLCCs are present in mechanical demanding tissues, such as skeletal tissue, however all three lysyl hydroxylases are expressed in the heart as well [36,37].

Similarly, LOX as well as two LOX-like proteins (LOXL-1 and LOXL-3) are highly expressed in the heart, while LOXL-2 is expressed during heart development [38]. There is solid evidence linking expression of LOX and LOXLs with fibrosis in various tissues [39]. In the heart, increased expression of LOX was found in hypertensive rats with LV hypertrophy, in mice after pressure overload and chronic HF and also in rats after myocardial infarction [[40], [41], [42]]. Moreover, increased LOX was reported in human patients with myocardial fibrosis due to hypertensive heart disease and chronic heart failure [43]. Therefore, LOX upregulation correlates with increased mechanical stiffness, as well as diastolic dysfunction due to excessive crosslinking [38]. Similarly, LOXL-2 is upregulated in diseased human hearts and elevated serum levels of LOXL-2 are found in patients with heart failure, suggesting it might be used as a biomarker for heart failure. Its levels correlate with collagen crosslinking and cardiac dysfunction. LOXL-2 inhibition or knock out improves and protects stress reduced cardiac dysfunction [44]. LOXL-1 was upregulated in cultured cardiomyocytes after treatment with hypertrophic stimuli and transgenic upregulation of LOX-1 led to cardiac hypertrophy, however the mechanism leading to this LOXL dependent hypertrophy is currently unclear [45]. Although there is currently no data directly implicating LHs in heart disease, one recent study implicated the long noncoding RNA wisper in cardiac fibrosis through association with the splicing factor TIAR, which then regulated the expression of LH2 [46].

### 3.2 Intracellular contributions

#### 3.2.1 Titin

The sarcomeric protein titin is the largest human protein. It spans from the *Z*-disc to the M-band (Fig. 1B). Titin has multiple critical function in the assembly and function of striated muscle sarcomeres ([47] for a recent review). It is a structural component and molecular ruler and is integral to muscle signalling, but also a molecular spring and primary contributor to the passive force in muscle. Recently it was suggested that it might also contribute to active force production, although this is still debated [47,48]. Titin's elasticity stems from the flexible I-band domain, including the N2B and PEKV spring elements. These flexible domains can be differentially expressed in the adult heart as either N2BA or N2B titin, whereby N2BA is longer and softer [[49], [50], [51], [52]]. The splicing factor RNA Binding Motif-20 (RBM20) represses splicing in a dose dependent way, leading to increased compliance [53,54]. A foetal N2BA isoform is even longer with additional spring elements, further reducing the stiffness. During postnatal development N2BA expression is reduced and N2B isoforms increase, thereby increasing the passive stiffness of the heart [52]. Importantly, titin's stiffness can be also modulated through phosphorylation, calcium binding, disulphide bridges or by interaction of the PEVK domain with actin [47], which is released with increasing calcium levels during the contraction cycle [55,56]. Noteworthy, several kinases that are known players in mechanosignalling, such as PKC and PKA can modulate titin stiffness. PKA (as well as PKG) phosphorylates the N2B element, reducing the stiffness. PKC on the other hand phosphorylates the PEVK-region, increasing the stiffness [51,57].

Titin truncation variants have been associated with dilated cardiomyopathy and have been found in up to 27% of patients with DCM, making it the largest-known genetic contribution to DCM [58]. Changes to titin isoform ratios have been also found in cardiac diseases. Increased (compliant) N2BA titin was detected in mice with pathological hypertrophy [59] as well as human patients with coronary artery disease [60], patients with heart failure with preserved ejection fraction [61], or end stage heart failure due to dilated cardiomyopathy [62,63], where changes to the stiffness in the heart have been observed.

#### 3.2.2 Microtubules

In addition to titin, the extrasarcomeric cytoskeleton, especially the microtubule network together with intermediate filaments contribute to the regulation of the cardiomyocyte mechanics and mechanosensing (Fig. 1C). Microtubules consist of α/β-tubulin heterodimers and are highly dynamic structures, involved in mitosis, cell motility, or intracellular trafficking. Because of their viscoelastic properties, they provide resistance to deforming stresses, which is enhanced through crosslinking with actin and intermediate filaments to resist buckling [[64], [65], [66]]. Their contribution to cardiomyocyte mechanics has been first noted in the 90s, where increased free and polymerised β-tubulin were found in right ventricular pressure overload in a cat model [67]. Sarcomeric shortening extent and velocity, initially reduced after the pressure overload were normalised after depolymerisation of the microtubules, while treatment with taxol, which enhances polymerisation, reduced these measures in healthy cardiomyocytes. The authors suggested that the microtubules imposed a resistive intracellular load on the shortening. The increased resistive load in hypertrophied cells however impeded sarcomere motion [67]. After some controversy regarding these results, work from the Prosser lab more recently found that the resistance to compressive load in cardiomyocytes depends not only on the number of microtubules, but largely on posttranslational modification, specifically the detryosination of the microtubules [68]. Detryosinated tubulin forms crosslinks with the intermediate filament protein desmin at the *Z*-disc, which prevent low resistance sliding of the microtubules between the sarcomeres and causes high-resistance buckling during the cardiomyocyte contractions. Therefore, detryosination reduces the cardiomyocyte stiffness and viscoelasticity. Further, reversing the detryosination through overexpression of the tubulin tyrosine ligase (TTL) increased magnitude and velocity of sarcomeric shortening [68]. Although the exact enzyme, responsible for the detryosination in cardiomyocytes is still not known, a complex of vasohibin-1 (VASH1) with the small vasohibin binding protein (SVBP) were showing robust tubulin carboxypeptidase (TCP) activity in neurons [69]. VASH1 is also expressed abundantly in the heart, making it a likely candidate for TCP activity in cardiomyocytes.

The cardiomyocyte microtubule network is highly proliferated and detyrosinated in heart failure patients with DCM, ICM or HCM (if also [68,70]. Fittingly, isolated cardiomyocytes from these patients were also more viscoelastic than non-failing cardiomyocytes and contraction dynamics could be improved in these cells after colchicine treatment [70]. Notably, detyrosinated microtubules are also involved in mechanosignalling, as they were found to be required for stretch dependent reactive oxygen species production, which in turn sensitised the ryanoidine receptor to trigger calcium sparks [71,72].

#### 3.2.3 Intermediate filaments

Intermediate filaments are flexible, elastic and highly extensible, compared to the actin cytoskeleton or microtubules (persistence length typically <1 μm, compared to microtubules >1 mm). They strengthen under strain, act as load bearing springs and regulate cellular stiffness in non-muscle cells [73]. In striated muscle, intermediate filaments contribute to the passive stiffness of the sarcomere especially at shorter sarcomere lengths between 1.9 and 2.1 μm, approximately the working range of cardiac muscle [26,74,75]. Desmin is the main IF protein in cardiac tissue. In addition, vimentin (only found in fibroblasts and vascular cells), paranemin and sycoilin, syemenin, nestin and nuclear lamins are expressed in the heart, whereby nuclear lamins play an important role in the regulation of nuclear stiffness and lamin isoform expression correlates with tissue stiffness (e.g. discussed in a recent commentary [76]). Desmin is one of the first muscle specific proteins during embryonic development and is found in the murine cardiac tube around day 8 [77]. In line with the contribution to the passive stiffness, desmin has been postulated and demonstrated to counteract external stresses, potentially helping to keep sarcomeres in register during the development of hypertrophy [78]. In line with this, desmin mRNA levels were increased in hypertrophic guinea pig hearts and increased desmin (as well as disorganisation of filaments) were detected in explanted failing human myocardium [78]. Moreover, a two-fold increase in serine phosphorylation of desmin was detected in rat ischemic HF. These include at least one conserved serine at the head domain (Ser60 in human and rat, Ser50 in chick) that is phosphorylated by PKA, whereby phosphorylation leads to reduced filament integrity or formation [79,80] (Fig. 1C). Noteworthy however, no changes to desmin levels were detected in cats or dogs [78]. In agreement with the function as load bearing spring, desmin knockout mice develop muscle weakness in cardiac and skeletal muscle, presumably due to the increased susceptibility to physical strain during muscle contraction [81,82].

The cellular stiffness was significantly increased in primary muscle cells from patients with a heterozygous R350P mutation, which leads to age-dependent desmin-positive protein aggregation pathology, skeletal muscle weakness, dilated cardiomyopathy, as well as cardiac arrhythmias and conduction defects [73,81]. These cells were also more susceptible to cell stretch, resulting in increased cell death. Because the experiments were only performed in myoblasts, it is however unclear if or how these data would translate into heart muscle cells.

#### 3.2.4 Actomyosin

Actin and myosin are primarily involved in active contractile force production in cardiomyocytes, as integral part of the thin and thick filaments, respectively. There are however at least two ways in which actomyosin can contribute to the passive stiffness, which both seem to be mostly of relevance in diseased conditions. On one hand residual crossbridge formation was reported to increase the passive stiffness in hypertrophied hearts after isoproterenol treatment, albeit measurements were only performed in transverse direction by AFM [83]. On the other hand, our own work indicated that non-muscle isoforms of myosin and potentially also actin (or at least nucleators that only nucleate cytoplasmic actin [84]) are enhanced after hypertrophic stimulation, whereby the increased activity of non-muscle myosin depended on PKC and Src. Traction force measurements of primary neonatal rat cardiomyocytes with nanopillars demonstrated that enhanced non-muscle myosin activity was directly related to increased resting tension and further resulted in changes to the downstream mechanosensing through talin (Fig. 1D) [7]. Although active non-muscle myosin was also enhanced in disease models (myocardial infarction and DCM), where it localised to costameres, further experiments are still needed to investigate this mechanism in vivo.

## 4 The changing mechanosensing toolkit

The sensing of mechanical signals requires cell surface receptors and associated proteins, which are typically linked to the cytoskeleton on the intracellular side to balance the forces from the ECM or apply forces to probe the extracellular environment [85,86]. Mechanoreceptor binding affinities change in response to extra and intracellular forces. Moreover, adaptor proteins at the intracellular side may be displaced or deformed in reaction to the forces, resulting in the initiation of intracellular signalling cascades and leading to the change in cellular behaviour [1,6]. That may be in the form of modulating the stiffness of proteins such a titin (see above), modulating the ion flux across the cardiac cell membrane to trigger and modulate cyclic contraction and/or regulating gene expression. The placing of mechanosensors at adhesion sites allows them to sense subtle changes to intracellular or extracellular force and enable the cell to adapt to these changes. The literature however more and more points to the fact that disease signalling pathways can lead to a change not only of ECM proteins (and the stiffness of the ECM) but also many key receptors or mechanosensitive adaptors change isoforms or expression levels. These changes might be compensatory initially, but can lead to suboptimal binding affinities between different binding partners, or further downstream regulatory effects. In the next section we will therefore look at the key mechanosensing toolkit and how these components are implicated in various heart conditions.

### 4.1 Mechanoreceptors

The main receptors associated with the mechanical signalling are integrins, a large heterodimeric family of cell surface receptors. Integrins are capable of bidirectional signalling, i.e. they can take extracellular information and relay that internally or in response to various intracellular changes alter their interactions with the surrounding matrix. Despite their central role in force transmission and the detection of changes in loads placed upon the cell, integrins themselves lack catalytic activity required to directly convert mechanical inputs into chemical signals. Instead integrins interact with various other proteins at the adhesion complexes, which do have the required activity, including kinases, such as FAK, Src or Fyn, or mechanosensitive adaptor proteins, such as talin or filamin [87].

18 α and 8 β integrin subunits can be combined in 24 known ways in different cell types, though complexity is further added due to differential spicing of integrin encoding transcripts leading to increased functional differences. The α subunits determine in big part the affinity towards extracellular ligands, such as the different laminins [32], while the β-cytoplasmic tail influences intracellular ligand interactions. Because integrins differ in mechanical strength (e.g. binding/unbinding rates under force) changes in integrin expression pattern have also implications on force generation, actin flow and integrin recruitment, although such mechanical data is still lacking for most integrin combinations [88,89].

As the stiffness of the heart changes during development, the expression of integrin types and isoforms change as well [[90], [91], [92]]. The embryonic heart is abundant in the laminin binding integrin α6 (with high affinity towards Laminin α5, α3 and α1), as well as the collagen binding integrin α1. Levels of the laminin binding α7 integrin increase (X1 and X2 isoforms, with strongest affinities towards the laminin α5 and α1 chains respectively) and the collagen binding α1 as well as the fibronectin binding α5 integrins are reduced between embryonic and adult cardiomyocytes [32,[90], [91], [92], [93]]. While α7 seems to be still the dominant isoform it is strongly downregulated and the α1, α3 and α5 isoforms are increased in hypertrophied and ischemic hearts [91,[94], [95], [96]]. A concomitantly reduced expression of Laminin α5 (judging from various GEO data sets: GSE57344 – human heart failure [96]; GDS2258 – pressure overload hypertrophy [97]; GDS488 - myocardial infarction model [98]), suggests lower strength of the integrin-laminin bonds and perhaps a shift towards the integrin-fibronectin interactions.

Similarly, there is a switch in β1 integrin expression from β1A in the embryonic heart to β1D in the postnatal heart, as well as increased expression of β1D in postnatal isolated primary cardiomyocytes [90,92]. A further increase in the expression of β1D integrin was detected in the mouse hearts after transverse aortic constriction [99]. In a rat MI model, β1(A and D) and β3 integrin expression was increased in the infarct zone during the first week and then levelled off [100]. Only β1D integrin was found in cardiomyocytes in this study however; the increase of β3 integrin was mainly detected on the peri-infarct vessels, while β1A was primarily increased on cardiac fibroblasts and inflammatory cells [100]. Moreover β1D expression in primary neonatal rat cardiomyocytes was susceptible to inflammation signals, such as TNF, resulting in a downregulation [100].

The levels of β1-integrin in the heart seem to influence the health of cardiomyocytes. Overexpression of β1A and D was previously linked to an enhanced hypertrophic response after phenylephrine stimulation in neonatal rat cardiomyocytes [101]. A deficiency of β1 integrin (β1 heterozygous knockout) on the other hand resulted in higher levels of cardiomyocyte apoptosis and poorer cardiac function after MI, thereby possibly contributing to heart failure [102,103].

Apart from changes to the ligand binding strength, binding partners at the intracellular tails can also vary between different integrins and even β1 isoforms. Such proteins include ICAP1, talin2, or the ryanoidine receptor 2, affecting integrin activation, ECM-cytoskeleton linkage and Ca2+ handling, respectively [[104], [105], [106]]. Importantly, changes to integrin expression also affects the cytoskeletal organisation, through downstream signalling molecules, including tyrosine kinases, such as Src and RhoGEFs and GAPs [7,88,[107], [108], [109], [110], [111], [112]].

**Fig. 2:**
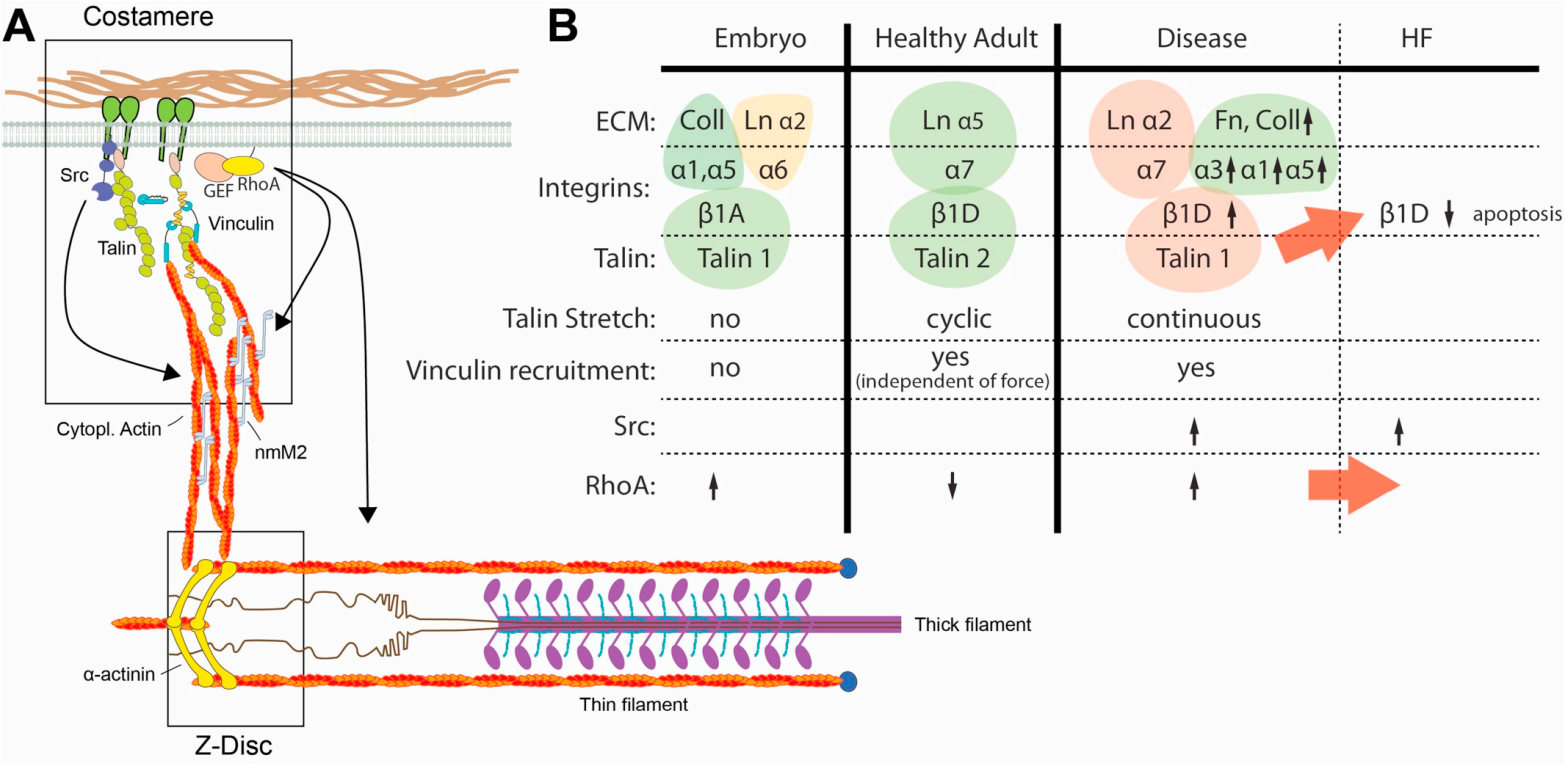
Changing components of the mechanosensing apparatus. A) Schematic of the integrin adhesions in the costameres and their connection to the sarcomere. Note only a few relevant components are displayed and proteins are not in scale. B) Overview of the different isoforms for ECM proteins, integrins and talin, as well as different downstream effects in embryonic, healthy adult and diseased hearts. Red arrows indicate events that can lead to progression to heart failure, i.e. the downregulation of β1D integrin through talin 1 overexpression, leading to apoptosis and heart failure; and heightened RhoA activity, which is also associated with heart failure. (For interpretation of the references to colour in this figure legend, the reader is referred to the web version of this article.)

Together this suggests that integrin expression is modified as an adaptive response to heart disease. However, because integrin adhesions are such prominent signalling hubs, these changes affect the mechanical signalling pathways and can lead to disease progression through a mismatch of ECM and integrin expression (Fig. 2) [113].

Besides integrins, recent research has identified mechanical signalling capabilities of cadherins at the cell-cell junctions, which, similar to integrins show catch bond behaviour (i.e. increasing the bond lifetime in reaction to force) and cytoskeletal tension plays an important role during the maturation of cadherin-mediated adhesions [114,115]. Additionally, the G-protein-coupled receptor-like protein Polycystin-1 senses stretch and after activation stabilises L-type calcium channels to enable a hypertrophic response [116]. Engagement of the hyaluronic acid (HA) receptor with its ligand can further augment mechanical signalling by integrins in cardiomyocytes in culture [117]. However, the presence of the HA receptor in cardiomyocytes is currently disputed. A previous study reported no prominent HA receptor staining in healthy rat atrial or ventricular cardiomyocytes in tissue sections [118]. On the other hand, another study found that osteopontin, an extracellular matrix protein involved in inflammation, which is significantly increased in the plasma of heart failure patients, interacted with the HA receptor in adult ventricular cardiomyocytes to induce apoptosis [119]. It is not clear from the study if the receptor was already expressed in the control cells. However, if osteopontin was causing the upregulation, the HA receptor could indeed play an important role in altering the mechanosignalling in cardiac disease. Also, mechanosensitive ion-channels, such as Piezo and especially TRP channels are expressed in cardiomyocytes and might be involved in pathological remodelling after myocardial infarction [120]. Similar to integrins, expression of these will differ between cell types, differentiation or disease state and therefore lead to altered mechanical responses. Moreover, not only the different mechanical signalling pathways are intertwined, they are also influenced and cross-react with growth factor or cytokine receptor signalling pathways, such as EGF signalling [121]. More research is however needed to fully understand how mechanical signals intersect with chemical signalling pathways and especially inflammation signalling in cardiac disease.

### 4.2 Mechanotransduction at the inner cell membrane

At the intracellular domains of integrins and other receptors are kinases and GTPases that initiate signalling cascades in response to the mechanical stimuli [6]. Moreover, bridging between the integrins and the cytoskeleton are various adapter proteins that influence the strength of the cytoskeletal coupling and feedback to the regulation of actin assembly and cytoskeletal organisation. Looking at all of them would be beyond the scope of this review article. However, amongst the adapter proteins talin stands out as a true mechanosignalling hub. Several recent studies have investigated the mechanosensitivity and isoform differences of talin in more detail, which we will discussed here, as this could be critical especially also for cardiomyocyte mechanosensing pathways.

Talin, a large (270 kDa) ubiquitously expressed protein is localised in cardiomyocytes at the costamere and intercalated disc. The protein itself consists of a head and tail arrangement. The head contains a FERM (protein 4.1, ezrin, radixin, moesin) domain, and is capable of binding directly to membranes containing phosphatidylinositol 4,5-bisphosphate (PIP2), binding and activating integrins in a form of inside-out signalling as discussed above, as well as binding to integrin associated proteins, including the GTPase RAP1, the Rho GTPase exchange factor TIAM1.

The majority of the talin molecule is composed of the C-terminal tail region and is subdivided into 11–13 bundles of 4–5 alpha helical structures in a rod like chain (R1- R13). The tail contains the actin binding sites (ABS) 2 and 3, which have distinct functions in mechanical sensing at least in non-muscle cells, where ABS2 is the main load bearing site and also required for stiffness sensing [122]. ABS3 in contrast is dispensable for rigidity sensing but required for spatial gradients in adhesion forces. Additionally the tail contains cryptic binding sites for vinculin on R2-R3, which become exposed under mechanical stretch, resulting in adhesion reinforcement [123].

Importantly however, recent in vitro, in silico and in cyto experiments have indicated that at physiological forces all talin rod domains can unfold, suggesting that this could influence the interaction with a range of different proteins, including kank, RIAM, or the Rho GTPase activating protein DLC1 [[123], [124], [125]]. Intriguingly, DLC1 is highly expressed in the heart, essential to heart development and potentially associated with congenital heart disease [126]. Our recent data showed different regimes of talin stretching depending on the ECM stiffness, with no stretching on embryonic, cyclic stretching on physiological stiffness and continuous stretching on fibrotic stiffness [7]. It is intriguing to hypothesise that this would affect the dynamics of talin binding partners to influence e.g. cytoskeletal rearrangements via DLC1. Alternatively, it is possible that enhancement of the integrin-talin-actin link through recruitment of vinculin will feedback into Src activity and this way modulate cytoskeleton assembly and tension at the integrin adhesions [7,107].

There are two known talin isoforms; talin 1 and talin 2 with approximately 74% sequence homology. Talin 1 and 2 have different mechanosensing activities: vinculin is recruited to cryptic binding sites on talin 1 in response to force, but vinculin recruitment to talin 2 occurs even in absence of force [127]. Additionally, talin 2 has a higher affinity for β1 and especially β1D integrin, which could be traced to a single residue in the F3 domain (C336 on talin 1, S339 on talin 2) [105,128]. Talin 1 is expressed in the embryonic heart and downregulated after birth, making talin 2 the primary isoform in the adult heart [129]. There appears to be an expressional co-regulation of the talin isoforms in the myocardium (although this was not the case in all cell types [128]), i.e. talin 1 reduction leads to talin 2 overexpression and vice versa [129,130].

Talin 1 expression is upregulated after aortic constriction and in heart failure patients. Although no significant change in talin 2 levels was seen in this study, this was nevertheless shifting the total ratio towards talin 1. Since β1D integrin was increased in the hypertrophic response this would lead to a further mismatch between the different components in the mechanotransduction pathway.

Talin 1 knockout mice have normal cardiac function and structure but show improved cardiac function after pressure overload. Knockout of talin 2 shows no impact on the heart on its own, perhaps due to the compensatory increase in talin 1 expression. The animals show strongly reduced β1D levels in the myocardium but relative activation of β1D is enhanced, leading to overall only a slight reduction in active β1 integrins [130]. This intriguing result suggests that the stronger binding of talin 2 might be required for the integrin stability or turnover, but talin 1 can ensure a minimum required amount of active integrin, possibly by being a stronger integrin activator. Loss of both isoforms together, however leads to displacement of vinculin from the costameres, increased membrane permeability and dilated cardiomyopathy [130].

### 4.3 Signalling to the nucleus and the role of the cytoskeleton

To change cellular behaviour, signalling proteins such as the kinases p38 or JNK or transcription factors such as NFkB shuttle to the nucleus at the end of various signalling cascades. These are normally retained in the cytoplasm by anchoring proteins, but released after modification of the anchoring proteins, typically through phosphorylation.

From the perspective of mechanosignalling, a particularly interesting group of these nuclear shuttling proteins is anchored by the actin cytoskeleton. This means that changes to the organisation of the cytoskeleton is not only outcome of mechanosensing; Because various regulators of actin assembly, crosslinking, severing and capping are direct or indirect downstream effectors of integrin, cytokine, Ca2+, ROS or other signals, this also provides a platform for communication between various signalling pathways. Actin assembly proteins compete for a limited pool of actin monomers, therefore increased activity of one will affect the activity of other actin assembly proteins with a preference for different actin structures, or different actin nucleation, elongation or bundling activities. This is therefore expected to have an effect on cytoplasmic retention or nuclear translocation of cytoskeleton anchored transcription factors.

The first protein where such mechanism was identified was myocardin-related transcription factor (MRTF) also known as MAL or MKF1 [131]. MRTF is anchored in the cytoplasm by G-actin. Competition with WH2 domains for G-actin dissociates the repressive complex, allowing activation and nuclear shuttling [132]. Because many WH2 domain proteins are actin assembly proteins (e.g. Leiomodin, or Spire) or nucleation promotion factors (e.g. WASP), this links actin assembly downstream of stretch or humoral stimulation to MRTF translocation to the nucleus, where it acts as co-factor for serum response factor (SRF) driven transcription [132,133]. However, because many genes of the actin cytoskeleton (including WH2 domain proteins, such as Leiomodin [134]) are expressed under the control of the SRF promoter, this creates a feed-forward loop [131]. Unsurprisingly, considering the key role of SRF driven genes in cardiomyocytes, simultaneous loss of MRTF-A and B leads to severe heart defects, including sarcomeric disarray and adult onset heart failure [135].

Two closely related transcription factors, YAP and TAZ, generally referred to as YAP/TAZ (although there might be cell and tissue specific differences in the influence of one vs the other [136]) have recently gained increasing attention. YAP/TAZ are the terminal effectors of the hippo signalling pathway and act as transcriptional co-factors with TEAD, SMAD 1–3 and other DNA binding proteins to regulate cell survival and proliferation and through that organ development and size. The recent interest is however at least in part related to a non-canonical hippo signalling role of YAP/TAZ in cellular mechanics, which is of relevance in a range of diseases, including cancer, atherosclerosis or fibrosis [137]. Similar to MRTF, YAP/TAZ is regulated by actin, however in this case it is the actin filaments and more specifically their conformation and tension that influences the nuclear translocation. The Rho GTPases RhoA and Rac1, the RhoGEF β-PIX, as well as actin filament capping or severing have all been shown to impact on the subcellular localisation of YAP/TAZ, e.g. downstream of integrin signalling. Moreover, direct forces on the nucleus, e.g. through the nuclear actin cap can speed up and increase the nuclear transport of YAP/TAZ [138,139]. The hippo pathway is also involved in heart development, cardiomyocyte apoptosis after myocardial infarction, and hypertrophic as well as dilated cardiomyopathies, which however seem to depend on MST and Lats and therefore, the canonical hippo signalling pathway rather than cytoskeletal arrangements [140].

In addition to MRTF and YAP/TAZ, several members of the family of LIM domain proteins are expressed in the heart and have been implicated in shuttling between the (cardiomyocyte) cytoskeleton and the nucleus in response to mechanical signals [141].

Amongst these proteins is Muscle LIM Protein (MLP/CSRP3), which has long been associated with cardiac disease. MLP KO mice develop a dilated cardiomyopathy phenotype and are staple model of the disease [142]. MLP also has a well-established link to DCM and HCM [143] in humans. Like most LIM proteins MLP is a protein binding protein with a wide interactome including cytoskeletal and adhesion proteins including actin, ILK, zyxin and T-cap/Telethonin as well as transcription factors such as MyoD and HDAC4 [144,145]. There has been much debate as to the nature of the role MLP plays in cardiomyocytes with disagreements in the literature over its subcellular localisation and overall function. Early work proposed that MLP functions as a mechanosensor at the *Z*-disc [146], however, more recent work indicated much broader localisation patterns [145,147]. MLP nuclear translocation is controlled in response to hypertrophic stimuli or electrical pacing and regulated by haem oxygenase 1 (which is regulated amongst others through Ca2+, is anti-hypertrophic and upregulated in the failing heart [148]) and HDAC4 [149,150], although the exact mechanism of the pathway is still elusive. Uniaxial cyclic stretching in contrast blocked nuclear accumulation, indicating that a balance between active contractile forces and passive tension is regulating the MLP targeting [149].

Recent work has demonstrated that MLP can negatively regulate PKCα activity in cardiomyocytes and indeed the loss of MLP leads to aberrant PKCα signalling in DCM [151]. Interestingly MLP has also been shown to be a substrate for PKCα, and that hyperphosphorylation is associated with DCM whilst hypophosphorylation is associated with HCM [151]. The intriguing correlation between MLP phosphorylation status and cardiomyopathy phenotype may be a further gateway into understanding this enigmatic protein.

FHL1 and 2 are two so-called LIM only proteins, both containing four and a half LIM domains, which both localise to the sarcomere [141]. In non-muscle cells, FHL2 moves to the nucleus in an actomyosin, force and stiffness dependent way that depends on focal adhesion kinase activity and its phosphorylation of tyrosine 93 on FHL2 [152]. The role in cardiac mechanosignalling is currently however disputed and to our knowledge no nuclear localisation has been reported in cardiomyocytes so far. Moreover, FHL2 deficient mice maintain a normal hypertrophic response after aortic constriction [153,154]. In response to adrenergic stress however, FHL2 is upregulated, but this blunts the hypertrophic response, indicating a cardioprotective role, which is further supported by clinical cases of cardiomyopathy with loss of functional FHL2 [154,155]. FHL1 functions as a scaffold for MAPK/ERK signalling and is activated in response to hypertrophic stimuli. It locates to the N2B region of titin and is thought to modulate the passive stretching of titin and thereby cardiac compliance [156,157].

Additionally, the family of muscle ankyrin repeat proteins (MARPs), including the members CARP1, CARP2 and CARP3 are able to shuttle between the cytoplasm and the nucleus, where they act as modulators for different transcription factors [158]. In the cytoplasm, they bind to the titin N2A region and other cytoplasmic proteins, noteworthy including talin and desmin in case of CARP1 [159,160]. MARPs change their localisation in response to stretch, which could be potentially due to differentially stretched titin N2A, or talin [158,161].

Lastly, the titin kinase domain (even though the kinase activity is debated and it might actually be a pseudokinase [162]) can be mechanically stretched, leading to a change in muscle gene regulation through an associated signalling complex. Titin kinase interacts with the autophagy cargo receptors nbr1 and p62/SQSTM1 (in a heterocomplex), while p62 in turn binds to MuRF2, a E3 ubiquitin ligase [163]. Intriguingly, arrest of beating resulted in dissociation of p62 from the sarcomeres and nuclear accumulation of MuRF2 and depletion of nuclear SRF, as well as reduced expression of SRF-controlled muscle genes.

## 5 Conclusions

Together, a role for the cytoskeleton beyond its structural/mechanical roles is becoming increasingly evident. Actin assembly is influenced by mechano- and other signalling pathways and through alteration of the assembly rates, or configuration of the actin structures it can provide a platform for further signalling to the nucleus as well as integration of multiple signalling pathways.

Importantly, the proteins and signalling mechanisms we have discussed here are by no means meant to be a definitive or exhaustive list of those involved in cardiomyocyte mechanobiology. As a field it is fair to say that cardiomyocyte mechanobiology has grown significantly but many questions remain open, in part due to difficulties to integrate data from in vivo models (often including expression data from non-cardiomyocytes) with results from 2D cell culture. However new techniques based on stem cells and genetic engineering as well as engineered heart tissues or lab-on-a-chip devices have the potential to bridge this gap and enable answering questions that were previously elusive.

## Transparency document

Transparency document. Image 1

## References

[1] Iskratsch T., Wolfenson H., Sheetz M.P. (2014). Appreciating force and shape-the rise of mechanotransduction in cell biology. Nat. Rev. Mol. Cell Biol..

[2] Emery J.L., Mithal A. (1961). Weights of cardiac ventricles at and after birth. Br. Heart J..

[3] Romero T., Covell J., Friedman W.F. (1972). A comparison of pressure-volume relations of the fetal, newborn, and adult heart. Am. J. Physiol..

[4] Rudolph A.M. (1970). The changes in the circulation after birth. Circulation.

[5] Andres-Delgado L., Mercader N. (2016). Interplay between cardiac function and heart development. Biochim. Biophys. Acta.

[6] Roca-Cusachs P., Iskratsch T., Sheetz M.P. (2012). Finding the weakest link: exploring integrin-mediated mechanical molecular pathways. J. Cell Sci..

[7] Pandey P., Hawkes W., Hu J., Megone W.V., Gautrot J., Anilkumar N., Zhang M., Hirvonen L., Cox S., Ehler E., Hone J., Sheetz M., Iskratsch T. (2018). Cardiomyocytes sense matrix rigidity through a combination of muscle and non-muscle myosin contractions. Dev. Cell.

[8] Lindsey S.E., Butcher J.T. (2011). The cycle of form and function in cardiac valvulogenesis. Aswan Heart Centre Science & Practice Series.

[9] Johnson P., Maxwell D.J., Tynan M.J., Allan L.D. (2000). Intracardiac pressures in the human fetus. Heart.

[10] McNally E.M., Golbus J.R., Puckelwartz M.J. (2013). Genetic mutations and mechanisms in dilated cardiomyopathy. J. Clin. Invest..

[11] Sanderson J.E. (2007). Heart failure with a normal ejection fraction. Heart.

[12] Yamamoto K., Masuyama T., Sakata Y., Doi R., Ono K., Mano T., Kondo H., Kuzuya T., Miwa T., Hori M. (2000). Local neurohumoral regulation in the transition to isolated diastolic heart failure in hypertensive heart disease: absence of AT1 receptor downregulation and 'overdrive' of the endothelin system. Cardiovasc. Res..

[13] Masuyama T., Yamamoto K., Sakata Y., Doi R., Nishikawa N., Kondo H., Ono K., Kuzuya T., Sugawara M., Hori M. (2000). Evolving changes in Doppler mitral flow velocity pattern in rats with hypertensive hypertrophy. J. Am. Coll. Cardiol..

[14] Roe A.T., Aronsen J.M., Skardal K., Hamdani N., Linke W.A., Danielsen H.E., Sejersted O.M., Sjaastad I., Louch W.E. (2017). Increased passive stiffness promotes diastolic dysfunction despite improved Ca2+ handling during left ventricular concentric hypertrophy. Cardiovasc. Res..

[15] Majkut S., Idema T., Swift J., Krieger C., Liu A., Discher D.E. (2013). Heart-specific stiffening in early embryos parallels matrix and myosin expression to optimize beating. Curr. Biol..

[16] Bhana B., Iyer R.K., Chen W.L., Zhao R., Sider K.L., Likhitpanichkul M., Simmons C.A., Radisic M. (2010). Influence of substrate stiffness on the phenotype of heart cells. Biotechnol. Bioeng..

[17] Berry M.F., Engler A.J., Woo Y.J., Pirolli T.J., Bish L.T., Jayasankar V., Morine K.J., Gardner T.J., Discher D.E., Sweeney H.L. (2006). Mesenchymal stem cell injection after myocardial infarction improves myocardial compliance. Am. J. Physiol. Heart Circ. Physiol..

[18] Kwiecinski W., Bessiere F., Colas E.C., N'Djin W.A., Tanter M., Lafon C., Pernot M. (2015). Cardiac shear-wave elastography using a transesophageal transducer: application to the mapping of thermal lesions in ultrasound transesophageal cardiac ablation. Phys. Med. Biol..

[19] Kolipaka A., Araoz P.A., McGee K.P., Manduca A., Ehman R.L. (2010). Magnetic resonance elastography as a method for the assessment of effective myocardial stiffness throughout the cardiac cycle. Magn. Reson. Med..

[20] Mazumder R., Schroeder S., Mo X., Litsky A.S., Clymer B.D., White R.D., Kolipaka A. (2017). In vivo magnetic resonance elastography to estimate left ventricular stiffness in a myocardial infarction induced porcine model. J. Magn. Reson. Imaging.

[21] Kolipaka A., McGee K., Aggarwal S., Anavekar N., Manduca A., Ehman R., Araoz P. (2011). A feasibility study: MR elastography as a method to compare stiffness estimates in hypertrophic obstructive cardiomyopathy and in normal volunteers. Proceedings of the 19th Annual Meeting of ISMRM, Montreal, Canada.

[22] Arani A., Arunachalam S.P., Chang I.C.Y., Baffour F., Rossman P.J., Glaser K.J., Trzasko J.D., McGee K.P., Manduca A., Grogan M., Dispenzieri A., Ehman R.L., Araoz P.A. (2017). Cardiac MR elastography for quantitative assessment of elevated myocardial stiffness in cardiac amyloidosis. J. Magn. Reson. Imaging.

[23] Hu Z., Metaxas D., Axel L. (2003). In vivo strain and stress estimation of the heart left and right ventricles from MRI images. Med. Image Anal..

[24] Sugimoto M., Oka H., Kajihama A., Nakau K., Kuwata S., Kurishima C., Azuma H. (2016). Non-invasive assessment of liver fibrosis by magnetic resonance elastography in patients with congenital heart disease undergoing the Fontan procedure and intracardiac repair. J. Cardiol..

[25] Glenn N.O., McKane M., Kohli V., Wen K.K., Rubenstein P.A., Bartman T., Sumanas S. (2012). The W-loop of alpha-cardiac actin is critical for heart function and endocardial cushion morphogenesis in zebrafish. Mol. Cell. Biol..

[26] Granzier H.L., Irving T.C. (1995). Passive tension in cardiac muscle: contribution of collagen, titin, microtubules, and intermediate filaments. Biophys. J..

[27] Lockhart M., Wirrig E., Phelps A., Wessels A. (2011). Extracellular matrix and heart development. Birth Defects Res. A Clin. Mol. Teratol..

[28] Rienks M., Papageorgiou A.P., Frangogiannis N.G., Heymans S. (2014). Myocardial extracellular matrix: an ever-changing and diverse entity. Circ. Res..

[29] Bishop J.E., Laurent G.J. (1995). Collagen turnover and its regulation in the normal and hypertrophying heart. Eur. Heart J..

[30] Oliviero P., Chassagne C., Salichon N., Corbier A., Hamon G., Marotte F., Charlemagne D., Rappaport L., Samuel J.L. (2000). Expression of laminin alpha2 chain during normal and pathological growth of myocardium in rat and human. Cardiovasc. Res..

[31] Miner J.H., Patton B.L., Lentz S.I., Gilbert D.J., Snider W.D., Jenkins N.A., Copeland N.G., Sanes J.R. (1997). The laminin alpha chains: expression, developmental transitions, and chromosomal locations of alpha1-5, identification of heterotrimeric laminins 8-11, and cloning of a novel alpha3 isoform. J. Cell Biol..

[32] Nishiuchi R., Takagi J., Hayashi M., Ido H., Yagi Y., Sanzen N., Tsuji T., Yamada M., Sekiguchi K. (2006). Ligand-binding specificities of laminin-binding integrins: a comprehensive survey of laminin-integrin interactions using recombinant alpha3beta1, alpha6beta1, alpha7beta1 and alpha6beta4 integrins. Matrix Biol..

[33] Marijianowski M.M., van der Loos C.M., Mohrschladt M.F., Becker A.E. (1994). The neonatal heart has a relatively high content of total collagen and type I collagen, a condition that may explain the less compliant state. J. Am. Coll. Cardiol..

[34] McCormick R.J., Thomas D.P. (1998). Collagen crosslinking in the heart: relationship to development and function. BAM-PADOVA.

[35] Reiser K., McCormick R.J., Rucker R.B. (1992). Enzymatic and nonenzymatic cross-linking of collagen and elastin. FASEB J..

[36] Yamauchi M., Sricholpech M. (2012). Lysine post-translational modifications of collagen. Essays Biochem..

[37] Ruotsalainen H., Sipila L., Kerkela E., Pospiech H., Myllyla R. (1999). Characterization of cDNAs for mouse lysyl hydroxylase 1, 2 and 3, their phylogenetic analysis and tissue-specific expression in the mouse. Matrix Biol..

[38] Lopez B., Gonzalez A., Hermida N., Valencia F., de Teresa E., Diez J. (2010). Role of lysyl oxidase in myocardial fibrosis: from basic science to clinical aspects. Am. J. Physiol. Heart Circ. Physiol..

[39] Maki J.M. (2009). Lysyl oxidases in mammalian development and certain pathological conditions. Histol. Histopathol..

[40] Hermida N., Lopez B., Gonzalez A., Dotor J., Lasarte J.J., Sarobe P., Borras-Cuesta F., Diez J. (2009). A synthetic peptide from transforming growth factor-beta1 type III receptor prevents myocardial fibrosis in spontaneously hypertensive rats. Cardiovasc. Res..

[41] Hughes W.M., Rodriguez W.E., Rosenberger D., Chen J., Sen U., Tyagi N., Moshal K.S., Vacek T., Kang Y.J., Tyagi S.C. (2008). Role of copper and homocysteine in pressure overload heart failure. Cardiovasc. Toxicol..

[42] McCormick R.J., Musch T.I., Bergman B.C., Thomas D.P. (1994). Regional differences in LV collagen accumulation and mature cross-linking after myocardial infarction in rats. Am. J. Phys..

[43] Lopez B., Querejeta R., Gonzalez A., Beaumont J., Larman M., Diez J. (2009). Impact of treatment on myocardial lysyl oxidase expression and collagen cross-linking in patients with heart failure. Hypertension.

[44] Yang J., Savvatis K., Kang J.S., Fan P., Zhong H., Schwartz K., Barry V., Mikels-Vigdal A., Karpinski S., Kornyeyev D., Adamkewicz J., Feng X., Zhou Q., Shang C., Kumar P., Phan D., Kasner M., Lopez B., Diez J., Wright K.C., Kovacs R.L., Chen P.S., Quertermous T., Smith V., Yao L., Tschope C., Chang C.P. (2016). Targeting LOXL2 for cardiac interstitial fibrosis and heart failure treatment. Nat. Commun..

[45] Ohmura H., Yasukawa H., Minami T., Sugi Y., Oba T., Nagata T., Kyogoku S., Ohshima H., Aoki H., Imaizumi T. (2012). Cardiomyocyte-specific transgenic expression of lysyl oxidase-like protein-1 induces cardiac hypertrophy in mice. Hypertens. Res..

[46] Micheletti R., Plaisance I., Abraham B.J., Sarre A., Ting C.C., Alexanian M., Maric D., Maison D., Nemir M., Young R.A., Schroen B., Gonzalez A., Ounzain S., Pedrazzini T. (2017). The long noncoding RNA Wisper controls cardiac fibrosis and remodeling. Sci. Transl. Med..

[47] Linke W.A. (2018). Titin gene and protein functions in passive and active muscle. Annu. Rev. Physiol..

[48] Rivas-Pardo J.A., Eckels E.C., Popa I., Kosuri P., Linke W.A., Fernandez J.M. (2016). Work done by titin protein folding assists muscle contraction. Cell Rep..

[49] Bang M.L., Centner T., Fornoff F., Geach A.J., Gotthardt M., McNabb M., Witt C.C., Labeit D., Gregorio C.C., Granzier H., Labeit S. (2001). The complete gene sequence of titin, expression of an unusual approximately 700-kDa titin isoform, and its interaction with obscurin identify a novel Z-line to I-band linking system. Circ. Res..

[50] Greaser M.L., Krzesinski P.R., Warren C.M., Kirkpatrick B., Campbell K.S., Moss R.L. (2005). Developmental changes in rat cardiac titin/connectin: transitions in normal animals and in mutants with a delayed pattern of isoform transition. J. Muscle Res. Cell Motil..

[51] Hidalgo C., Granzier H. (2013). Tuning the molecular giant titin through phosphorylation: role in health and disease. Trends Cardiovasc. Med..

[52] Lahmers S., Wu Y., Call D.R., Labeit S., Granzier H. (2004). Developmental control of titin isoform expression and passive stiffness in fetal and neonatal myocardium. Circ. Res..

[53] Guo W., Schafer S., Greaser M.L., Radke M.H., Liss M., Govindarajan T., Maatz H., Schulz H., Li S., Parrish A.M., Dauksaite V., Vakeel P., Klaassen S., Gerull B., Thierfelder L., Regitz-Zagrosek V., Hacker T.A., Saupe K.W., Dec G.W., Ellinor P.T., MacRae C.A., Spallek B., Fischer R., Perrot A., Ozcelik C., Saar K., Hubner N., Gotthardt M. (2012). RBM20, a gene for hereditary cardiomyopathy, regulates titin splicing. Nat. Med..

[54] Methawasin M., Strom J.G., Slater R.E., Fernandez V., Saripalli C., Granzier H. (2016). Experimentally increasing the compliance of titin through RNA binding Motif-20 (RBM20) inhibition improves diastolic function in a mouse model of heart failure with preserved ejection fraction. Circulation.

[55] Kulke M., Fujita-Becker S., Rostkova E., Neagoe C., Labeit D., Manstein D.J., Gautel M., Linke W.A. (2001). Interaction between PEVK-titin and actin filaments: origin of a viscous force component in cardiac myofibrils. Circ. Res..

[56] Yamasaki R., Berri M., Wu Y., Trombitas K., McNabb M., Kellermayer M.S., Witt C., Labeit D., Labeit S., Greaser M., Granzier H. (2001). Titin-actin interaction in mouse myocardium: passive tension modulation and its regulation by calcium/S100A1. Biophys. J..

[57] Kotter S., Gout L., Von Frieling-Salewsky M., Muller A.E., Helling S., Marcus K., Dos Remedios C., Linke W.A., Kruger M. (2013). Differential changes in titin domain phosphorylation increase myofilament stiffness in failing human hearts. Cardiovasc. Res..

[58] Herman D.S., Lam L., Taylor M.R., Wang L., Teekakirikul P., Christodoulou D., Conner L., DePalma S.R., McDonough B., Sparks E., Teodorescu D.L., Cirino A.L., Banner N.R., Pennell D.J., Graw S., Merlo M., Di Lenarda A., Sinagra G., Bos J.M., Ackerman M.J., Mitchell R.N., Murry C.E., Lakdawala N.K., Ho C.Y., Barton P.J., Cook S.A., Mestroni L., Seidman J.G., Seidman C.E. (2012). Truncations of titin causing dilated cardiomyopathy. N. Engl. J. Med..

[59] Hudson B., Hidalgo C., Saripalli C., Granzier H. (2011). Hyperphosphorylation of mouse cardiac titin contributes to transverse aortic constriction-induced diastolic dysfunction. Circ. Res..

[60] Neagoe C., Kulke M., del Monte F., Gwathmey J.K., de Tombe P.P., Hajjar R.J., Linke W.A. (2002). Titin isoform switch in ischemic human heart disease. Circulation.

[61] Borbely A., Falcao-Pires I., van Heerebeek L., Hamdani N., Edes I., Gavina C., Leite-Moreira A.F., Bronzwaer J.G., Papp Z., van der Velden J., Stienen G.J., Paulus W.J. (2009). Hypophosphorylation of the stiff N2B titin isoform raises cardiomyocyte resting tension in failing human myocardium. Circ. Res..

[62] Makarenko I., Opitz C.A., Leake M.C., Neagoe C., Kulke M., Gwathmey J.K., del Monte F., Hajjar R.J., Linke W.A. (2004). Passive stiffness changes caused by upregulation of compliant titin isoforms in human dilated cardiomyopathy hearts. Circ. Res..

[63] Nagueh S.F., Shah G., Wu Y., Torre-Amione G., King N.M., Lahmers S., Witt C.C., Becker K., Labeit S., Granzier H.L. (2004). Altered titin expression, myocardial stiffness, and left ventricular function in patients with dilated cardiomyopathy. Circulation.

[64] Brangwynne C.P., MacKintosh F.C., Kumar S., Geisse N.A., Talbot J., Mahadevan L., Parker K.K., Ingber D.E., Weitz D.A. (2006). Microtubules can bear enhanced compressive loads in living cells because of lateral reinforcement. J. Cell Biol..

[65] Brodland G.W., Gordon R. (1990). Intermediate filaments may prevent buckling of compressively loaded microtubules. J. Biomech. Eng..

[66] Wang N., Butler J.P., Ingber D.E. (1993). Mechanotransduction across the cell surface and through the cytoskeleton. Science.

[67] Tsutsui H., Ishihara K., Cooper G.T. (1993). Cytoskeletal role in the contractile dysfunction of hypertrophied myocardium. Science.

[68] Robison P., Caporizzo M.A., Ahmadzadeh H., Bogush A.I., Chen C.Y., Margulies K.B., Shenoy V.B., Prosser B.L. (2016). Detyrosinated microtubules buckle and bear load in contracting cardiomyocytes. Science.

[69] Aillaud C., Bosc C., Peris L., Bosson A., Heemeryck P., Van Dijk J., Le Friec J., Boulan B., Vossier F., Sanman L.E., Syed S., Amara N., Coute Y., Lafanechere L., Denarier E., Delphin C., Pelletier L., Humbert S., Bogyo M., Andrieux A., Rogowski K., Moutin M.J. (2017). Vasohibins/SVBP are tubulin carboxypeptidases (TCPs) that regulate neuron differentiation. Science.

[70] Chen C.Y., Caporizzo M.A., Bedi K., Vite A., Bogush A.I., Robison P., Heffler J.G., Salomon A.K., Kelly N.A., Babu A., Morley M.P., Margulies K.B., Prosser B.L. (2018). Suppression of detyrosinated microtubules improves cardiomyocyte function in human heart failure. Nat. Med..

[71] Prosser B.L., Ward C.W., Lederer W.J. (2011). X-ROS signaling: rapid mechano-chemo transduction in heart. Science.

[72] Kerr J.P., Robison P., Shi G., Bogush A.I., Kempema A.M., Hexum J.K., Becerra N., Harki D.A., Martin S.S., Raiteri R., Prosser B.L., Ward C.W. (2015). Detyrosinated microtubules modulate mechanotransduction in heart and skeletal muscle. Nat. Commun..

[73] Clemen C.S., Herrmann H., Strelkov S.V., Schroder R. (2013). Desminopathies: pathology and mechanisms. Acta Neuropathol..

[74] Guccione J.M., O'Dell W.G., McCulloch A.D., Hunter W.C. (1997). Anterior and posterior left ventricular sarcomere lengths behave similarly during ejection. Am. J. Phys..

[75] Pollack G.H., Huntsman L.L. (1974). Sarcomere length-active force relations in living mammalian cardiac muscle. Am. J. Phys..

[76] Swift J., Discher D.E. (2014). The nuclear lamina is mechano-responsive to ECM elasticity in mature tissue. J. Cell Sci..

[77] Kuisk I.R., Li H., Tran D., Capetanaki Y. (1996). A single MEF2 site governs desmin transcription in both heart and skeletal muscle during mouse embryogenesis. Dev. Biol..

[78] Hein S., Kostin S., Heling A., Maeno Y., Schaper J. (2000). The role of the cytoskeleton in heart failure. Cardiovasc. Res..

[79] Geisler N., Weber K. (1988). Phosphorylation of desmin in vitro inhibits formation of intermediate filaments; identification of three kinase a sites in the aminoterminal head domain. EMBO J..

[80] Bouvet M., Dubois-Deruy E., Alayi T.D., Mulder P., El Amranii M., Beseme O., Amouyel P., Richard V., Tomavo S., Pinet F. (2016). Increased level of phosphorylated desmin and its degradation products in heart failure. Biochem. Biophys. Rep..

[81] Clemen C.S., Stockigt F., Strucksberg K.H., Chevessier F., Winter L., Schutz J., Bauer R., Thorweihe J.M., Wenzel D., Schlotzer-Schrehardt U., Rasche V., Krsmanovic P., Katus H.A., Rottbauer W., Just S., Muller O.J., Friedrich O., Meyer R., Herrmann H., Schrickel J.W., Schroder R. (2015). The toxic effect of R350P mutant desmin in striated muscle of man and mouse. Acta Neuropathol..

[82] Thornell L., Carlsson L., Li Z., Mericskay M., Paulin D. (1997). Null mutation in the desmin gene gives rise to a cardiomyopathy. J. Mol. Cell. Cardiol..

[83] Sumita Yoshikawa W., Nakamura K., Miura D., Shimizu J., Hashimoto K., Kataoka N., Toyota H., Okuyama H., Miyoshi T., Morita H., Fukushima Kusano K., Matsuo T., Takaki M., Kajiya F., Yagi N., Ohe T., Ito H. (2013). Increased passive stiffness of cardiomyocytes in the transverse direction and residual actin and myosin cross-bridge formation in hypertrophied rat hearts induced by chronic beta-adrenergic stimulation. Circ. J..

[84] Patel A.A., Oztug Durer Z.A., van Loon A.P., Bremer K.V., Quinlan M.E. (2017). Drosophila and human FHOD family formins nucleate actin filaments. J. Biol. Chem..

[85] Meacci G., Wolfenson H., Liu S., Stachowiak M.R., Iskratsch T., Mathur A., Ghassemi S., Gauthier N., Tabdanov E., Lohner J., Gondarenko A., Chander A.C., Roca-Cusachs P., O'Shaughnessy B., Hone J., Sheetz M.P. (2016). Alpha-actinin links ECM rigidity sensing contractile units with periodic cell edge retractions. Mol. Biol. Cell.

[86] Wolfenson H., Meacci G., Liu S., Stachowiak M.R., Iskratsch T., Ghassemi S., Roca-Cusachs P., O'Shaughnessy B., Hone J., Sheetz M.P. (2016). Tropomyosin controls sarcomere-like contractions for rigidity sensing and suppressing growth on soft matrices. Nat. Cell Biol..

[87] Legate K.R., Fassler R. (2009). Mechanisms that regulate adaptor binding to beta-integrin cytoplasmic tails. J. Cell Sci..

[88] Elosegui-Artola A., Bazellieres E., Allen M.D., Andreu I., Oria R., Sunyer R., Gomm J.J., Marshall J.F., Jones J.L., Trepat X., Roca-Cusachs P. (2014). Rigidity sensing and adaptation through regulation of integrin types. Nat. Mater..

[89] Bharadwaj M., Strohmeyer N., Colo G.P., Helenius J., Beerenwinkel N., Schiller H.B., Fassler R., Muller D.J. (2017). alphaV-class integrins exert dual roles on alpha5beta1 integrins to strengthen adhesion to fibronectin. Nat. Commun..

[90] Maitra N., Flink I.L., Bahl J.J., Morkin E. (2000). Expression of alpha and beta integrins during terminal differentiation of cardiomyocytes. Cardiovasc. Res..

[91] Terracio L., Rubin K., Gullberg D., Balog E., Carver W., Jyring R., Borg T.K. (1991). Expression of collagen binding integrins during cardiac development and hypertrophy. Circ. Res..

[92] Brancaccio M., Cabodi S., Belkin A.M., Collo G., Tomatis D., Altruda F., Silengo L., TARONE G. (1998). Differential onset of expression of α7 and β1D integrins during mouse heart and skeletal muscle development. Cell Adhes. Commun..

[93] Ziober B.L., Vu M.P., Waleh N., Crawford J., Lin C.S., Kramer R.H. (1993). Alternative extracellular and cytoplasmic domains of the integrin alpha 7 subunit are differentially expressed during development. J. Biol. Chem..

[94] Nawata J., Ohno I., Isoyama S., Suzuki J., Miura S., Ikeda J., Shirato K. (1999). Differential expression of alpha 1, alpha 3 and alpha 5 integrin subunits in acute and chronic stages of myocardial infarction in rats. Cardiovasc. Res..

[95] Brancaccio M., Hirsch E., Notte A., Selvetella G., Lembo G., Tarone G. (2006). Integrin signalling: the tug-of-war in heart hypertrophy. Cardiovasc. Res..

[96] Liu Y., Morley M., Brandimarto J., Hannenhalli S., Hu Y., Ashley E.A., Tang W.H., Moravec C.S., Margulies K.B., Cappola T.P., Li M., M.A. consortium (2015). RNA-Seq identifies novel myocardial gene expression signatures of heart failure. Genomics.

[97] Mirotsou M., Dzau V.J., Pratt R.E., Weinberg E.O. (2006). Physiological genomics of cardiac disease: quantitative relationships between gene expression and left ventricular hypertrophy. Physiol. Genomics.

[98] Tarnavski O., McMullen J.R., Schinke M., Nie Q., Kong S., Izumo S. (2004). Mouse cardiac surgery: comprehensive techniques for the generation of mouse models of human diseases and their application for genomic studies. Physiol. Genomics.

[99] Babbitt C.J., Shai S.Y., Harpf A.E., Pham C.G., Ross R.S. (2002). Modulation of integrins and integrin signaling molecules in the pressure-loaded murine ventricle. Histochem. Cell Biol..

[100] Sun M., Opavsky M.A., Stewart D.J., Rabinovitch M., Dawood F., Wen W.H., Liu P.P. (2003). Temporal response and localization of integrins beta1 and beta3 in the heart after myocardial infarction: regulation by cytokines. Circulation.

[101] Ross R.S., Pham C., Shai S.Y., Goldhaber J.I., Fenczik C., Glembotski C.C., Ginsberg M.H., Loftus J.C. (1998). Beta1 integrins participate in the hypertrophic response of rat ventricular myocytes. Circ. Res..

[102] Ding B., Price R.L., Goldsmith E.C., Borg T.K., Yan X., Douglas P.S., Weinberg E.O., Bartunek J., Thielen T., Didenko V.V., Lorell B.H. (2000). Left ventricular hypertrophy in ascending aortic stenosis mice: anoikis and the progression to early failure. Circulation.

[103] Krishnamurthy P., Subramanian V., Singh M., Singh K. (2006). Deficiency of beta1 integrins results in increased myocardial dysfunction after myocardial infarction. Heart.

[104] Okada H., Lai N.C., Kawaraguchi Y., Liao P., Copps J., Sugano Y., Okada-Maeda S., Banerjee I., Schilling J.M., Gingras A.R., Asfaw E.K., Suarez J., Kang S.M., Perkins G.A., Au C.G., Israeli-Rosenberg S., Manso A.M., Liu Z., Milner D.J., Kaufman S.J., Patel H.H., Roth D.M., Hammond H.K., Taylor S.S., Dillmann W.H., Goldhaber J.I., Ross R.S. (2013). Integrins protect cardiomyocytes from ischemia/reperfusion injury. J. Clin. Invest..

[105] Anthis N.J., Wegener K.L., Critchley D.R., Campbell I.D. (2010). Structural diversity in integrin/talin interactions. Structure.

[106] Millon-Fremillon A., Brunner M., Abed N., Collomb E., Ribba A.S., Block M.R., Albiges-Rizo C., Bouvard D. (2013). Calcium and calmodulin-dependent serine/threonine protein kinase type II (CaMKII)-mediated intramolecular opening of integrin cytoplasmic domain-associated protein-1 (ICAP-1alpha) negatively regulates beta1 integrins. J. Biol. Chem..

[107] Das M., Ithychanda S., Qin J., Plow E.F. (2014). Mechanisms of talin-dependent integrin signaling and crosstalk. Biochim. Biophys. Acta.

[108] Takefuji M., Kruger M., Sivaraj K.K., Kaibuchi K., Offermanns S., Wettschureck N. (2013). RhoGEF12 controls cardiac remodeling by integrating G protein- and integrin-dependent signaling cascades. J. Exp. Med..

[109] Iskratsch T., Yu C.H., Mathur A., Liu S., Stevenin V., Dwyer J., Hone J., Ehler E., Sheetz M. (2013). FHOD1 is needed for directed forces and adhesion maturation during cell spreading and migration. Dev. Cell.

[110] Tabdanov E., Gondarenko S., Kumari S., Liapis A., Dustin M.L., Sheetz M.P., Kam L.C., Iskratsch T. (2015). Micropatterning of TCR and LFA-1 ligands reveals complementary effects on cytoskeleton mechanics in T cells. Integr. Biol. (Camb.).

[111] Takeishi Y., Huang Q., Abe J., Glassman M., Che W., Lee J.D., Kawakatsu H., Lawrence E.G., Hoit B.D., Berk B.C., Walsh R.A. (2001). Src and multiple MAP kinase activation in cardiac hypertrophy and congestive heart failure under chronic pressure-overload: comparison with acute mechanical stretch. J. Mol. Cell. Cardiol..

[112] Wang S., Gong H., Jiang G., Ye Y., Wu J., You J., Zhang G., Sun A., Komuro I., Ge J., Zou Y. (2014). Src is required for mechanical stretch-induced cardiomyocyte hypertrophy through angiotensin II type 1 receptor-dependent beta-arrestin2 pathways. PLoS One.

[113] Ross R.S., Borg T.K. (2001). Integrins and the myocardium. Circ. Res..

[114] Hoffman B.D., Yap A.S. (2015). Towards a dynamic understanding of cadherin-based mechanobiology. Trends Cell Biol..

[115] Zaidel-Bar R. (2013). Cadherin adhesome at a glance. J. Cell Sci..

[116] Pedrozo Z., Criollo A., Battiprolu P.K., Morales C.R., Contreras-Ferrat A., Fernandez C., Jiang N., Luo X., Caplan M.J., Somlo S., Rothermel B.A., Gillette T.G., Lavandero S., Hill J.A. (2015). Polycystin-1 is a cardiomyocyte mechanosensor that governs L-type Ca2+ channel protein stability. Circulation.

[117] Chopra A., Murray M.E., Byfield F.J., Mendez M.G., Halleluyan R., Restle D.J., Raz-Ben Aroush D., Galie P.A., Pogoda K., Bucki R., Marcinkiewicz C., Prestwich G.D., Zarembinski T.I., Chen C.S., Pure E., Kresh J.Y., Janmey P.A. (2014). Augmentation of integrin-mediated mechanotransduction by hyaluronic acid. Biomaterials.

[118] Hellstrom M., Johansson B., Engstrom-Laurent A. (2006). Hyaluronan and its receptor CD44 in the heart of newborn and adult rats. Anat Rec A Discov Mol Cell Evol Biol.

[119] Dalal S., Zha Q., Daniels C.R., Steagall R.J., Joyner W.L., Gadeau A.P., Singh M., Singh K. (2014). Osteopontin stimulates apoptosis in adult cardiac myocytes via the involvement of CD44 receptors, mitochondrial death pathway, and endoplasmic reticulum stress. Am. J. Physiol. Heart Circ. Physiol..

[120] Peyronnet R., Nerbonne J.M., Kohl P. (2016). Cardiac mechano-gated ion channels and arrhythmias. Circ. Res..

[121] Saxena M., Liu S., Yang B., Hajal C., Changede R., Hu J., Wolfenson H., Hone J., Sheetz M.P. (2017). EGFR and HER2 activate rigidity sensing only on rigid matrices. Nat. Mater..

[122] Kumar A., Ouyang M., Van den Dries K., McGhee E.J., Tanaka K., Anderson M.D., Groisman A., Goult B.T., Anderson K.I., Schwartz M.A. (2016). Talin tension sensor reveals novel features of focal adhesion force transmission and mechanosensitivity. J. Cell Biol..

[123] Klapholz B., Brown N.H. (2017). Talin - the master of integrin adhesions. J. Cell Sci..

[124] Margadant F., Chew L.L., Hu X., Yu H., Bate N., Zhang X., Sheetz M. (2011). Mechanotransduction in vivo by repeated talin stretch-relaxation events depends upon vinculin. PLoS Biol..

[125] Yao M., Goult B.T., Klapholz B., Hu X., Toseland C.P., Guo Y., Cong P., Sheetz M.P., Yan J. (2016). The mechanical response of talin. Nat. Commun..

[126] Lin B., Wang Y., Wang Z., Tan H., Kong X., Shu Y., Zhang Y., Huang Y., Zhu Y., Xu H., Wang Z., Wang P., Ning G., Kong X., Hu G., Hu L. (2014). Uncovering the rare variants of DLC1 isoform 1 and their functional effects in a Chinese sporadic congenital heart disease cohort. PLoS One.

[127] Austen K., Ringer P., Mehlich A., Chrostek-Grashoff A., Kluger C., Klingner C., Sabass B., Zent R., Rief M., Grashoff C. (2015). Extracellular rigidity sensing by talin isoform-specific mechanical linkages. Nat. Cell Biol..

[128] Qi L., Jafari N., Li X., Chen Z., Li L., Hytonen V.P., Goult B.T., Zhan C.G., Huang C. (2016). Talin2-mediated traction force drives matrix degradation and cell invasion. J. Cell Sci..

[129] Manso A.M., Li R., Monkley S.J., Cruz N.M., Ong S., Lao D.H., Koshman Y.E., Gu Y., Peterson K.L., Chen J., Abel E.D., Samarel A.M., Critchley D.R., Ross R.S. (2013). Talin1 has unique expression versus talin 2 in the heart and modifies the hypertrophic response to pressure overload. J. Biol. Chem..

[130] Manso A.M., Okada H., Sakamoto F.M., Moreno E., Monkley S.J., Li R., Critchley D.R., Ross R.S. (2017). Loss of mouse cardiomyocyte talin-1 and talin-2 leads to beta-1 integrin reduction, costameric instability, and dilated cardiomyopathy. Proc. Natl. Acad. Sci. U. S. A..

[131] Posern G., Treisman R. (2006). Actin' together: serum response factor, its cofactors and the link to signal transduction. Trends Cell Biol..

[132] Weissbach J., Schikora F., Weber A., Kessels M., Posern G. (2016). Myocardin-related transcription factor A activation by competition with WH2 domain proteins for actin binding. Mol. Cell. Biol..

[133] Kuwahara K., Kinoshita H., Kuwabara Y., Nakagawa Y., Usami S., Minami T., Yamada Y., Fujiwara M., Nakao K. (2010). Myocardin-related transcription factor a is a common mediator of mechanical stress- and neurohumoral stimulation-induced cardiac hypertrophic signaling leading to activation of brain natriuretic peptide gene expression. Mol. Cell. Biol..

[134] Cenik B.K., Garg A., McAnally J.R., Shelton J.M., Richardson J.A., Bassel-Duby R., Olson E.N., Liu N. (2015). Severe myopathy in mice lacking the MEF2/SRF-dependent gene leiomodin-3. J. Clin. Invest..

[135] Mokalled M.H., Carroll K.J., Cenik B.K., Chen B., Liu N., Olson E.N., Bassel-Duby R. (2015). Myocardin-related transcription factors are required for cardiac development and function. Dev. Biol..

[136] Plouffe S.W., Lin K.C., Moore J.L., Tan F.E., Ma S., Ye Z., Qiu Y., Ren B., Guan K.L. (2018). The hippo pathway effector proteins YAP and TAZ have both distinct and overlapping functions in the cell. J. Biol. Chem..

[137] Totaro A., Panciera T., Piccolo S. (2018). YAP/TAZ upstream signals and downstream responses. Nat. Cell Biol..

[138] Elosegui-Artola A., Andreu I., Beedle A.E.M., Lezamiz A., Uroz M., Kosmalska A.J., Oria R., Kechagia J.Z., Rico-Lastres P., Le Roux A.L., Shanahan C.M., Trepat X., Navajas D., Garcia-Manyes S., Roca-Cusachs P. (2017). Force triggers YAP nuclear entry by regulating transport across nuclear pores. Cell.

[139] Shiu J.Y., Aires L., Lin Z., Vogel V. (2018). Nanopillar force measurements reveal actin-cap-mediated YAP mechanotransduction. Nat. Cell Biol..

[140] Zhou Q., Li L., Zhao B., Guan K.L. (2015). The hippo pathway in heart development, regeneration, and diseases. Circ. Res..

[141] Li A., Ponten F., dos Remedios C.G. (2012). The interactome of LIM domain proteins: the contributions of LIM domain proteins to heart failure and heart development. Proteomics.

[142] Arber S., Hunter J.J., Ross J., Hongo M., Sansig G., Borg J., Perriard J.C., Chien K.R., Caroni P. (1997). MLP-deficient mice exhibit a disruption of cardiac cytoarchitectural organization, dilated cardiomyopathy, and heart failure. Cell.

[143] Geier C., Perrot A., Ozcelik C., Binner P., Counsell D., Hoffmann K., Pilz B., Martiniak Y., Gehmlich K., van der Ven P.F., Furst D.O., Vornwald A., von Hodenberg E., Nurnberg P., Scheffold T., Dietz R., Osterziel K.J. (2003). Mutations in the human muscle LIM protein gene in families with hypertrophic cardiomyopathy. Circulation.

[144] Gehmlich K., Geier C., Milting H., Furst D., Ehler E. (2008). Back to square one: what do we know about the functions of muscle LIM protein in the heart?. J. Muscle Res. Cell Motil..

[145] Hoffmann C., Moreau F., Moes M., Luthold C., Dieterle M., Goretti E., Neumann K., Steinmetz A., Thomas C. (2014). Human muscle LIM protein dimerizes along the actin cytoskeleton and cross-links actin filaments. Mol. Cell. Biol..

[146] Knoll R., Hoshijima M., Hoffman H.M., Person V., Lorenzen-Schmidt I., Bang M.L., Hayashi T., Shiga N., Yasukawa H., Schaper W., McKenna W., Yokoyama M., Schork N.J., Omens J.H., McCulloch A.D., Kimura A., Gregorio C.C., Poller W., Schaper J., Schultheiss H.P., Chien K.R. (2002). The cardiac mechanical stretch sensor machinery involves a Z disc complex that is defective in a subset of human dilated cardiomyopathy. Cell.

[147] Boateng S.Y., Belin R.J., Geenen D.L., Margulies K.B., Martin J.L., Hoshijima M., de Tombe P.P., Russell B. (2007). Cardiac dysfunction and heart failure are associated with abnormalities in the subcellular distribution and amounts of oligomeric muscle LIM protein. Am. J. Physiol. Heart Circ. Physiol..

[148] Hu C.M., Chen Y.H., Chiang M.T., Chau L.Y. (2004). Heme oxygenase-1 inhibits angiotensin II-induced cardiac hypertrophy in vitro and in vivo. Circulation.

[149] Boateng S.Y., Senyo S.E., Qi L., Goldspink P.H., Russell B. (2009). Myocyte remodeling in response to hypertrophic stimuli requires nucleocytoplasmic shuttling of muscle LIM protein. J. Mol. Cell. Cardiol..

[150] Paudyal A., Dewan S., Ikie C., Whalley B.J., de Tombe P.P., Boateng S.Y. (2016). Nuclear accumulation of myocyte muscle LIM protein is regulated by heme oxygenase 1 and correlates with cardiac function in the transition to failure. J. Physiol..

[151] Lange S., Gehmlich K., Lun A.S., Blondelle J., Hooper C., Dalton N.D., Alvarez E.A., Zhang X., Bang M.L., Abassi Y.A., Dos Remedios C.G., Peterson K.L., Chen J., Ehler E. (2016). MLP and CARP are linked to chronic PKCalpha signalling in dilated cardiomyopathy. Nat. Commun..

[152] Nakazawa N., Sathe A.R., Shivashankar G.V., Sheetz M.P. (2016). Matrix mechanics controls FHL2 movement to the nucleus to activate p21 expression. Proc. Natl. Acad. Sci. U. S. A..

[153] Chu P.H., Bardwell W.M., Gu Y., Ross J., Chen J. (2000). FHL2 (SLIM3) is not essential for cardiac development and function. Mol. Cell. Biol..

[154] Hojayev B., Rothermel B.A., Gillette T.G., Hill J.A. (2012). FHL2 binds calcineurin and represses pathological cardiac growth. Mol. Cell. Biol..

[155] Friedrich F.W., Reischmann S., Schwalm A., Unger A., Ramanujam D., Munch J., Muller O.J., Hengstenberg C., Galve E., Charron P., Linke W.A., Engelhardt S., Patten M., Richard P., van der Velden J., Eschenhagen T., Isnard R., Carrier L. (2014). FHL2 expression and variants in hypertrophic cardiomyopathy. Basic Res. Cardiol..

[156] Sheikh F., Raskin A., Chu P.H., Lange S., Domenighetti A.A., Zheng M., Liang X., Zhang T., Yajima T., Gu Y., Dalton N.D., Mahata S.K., Dorn G.W., Brown J.H., Peterson K.L., Omens J.H., McCulloch A.D., Chen J. (2008). An FHL1-containing complex within the cardiomyocyte sarcomere mediates hypertrophic biomechanical stress responses in mice. J. Clin. Invest..

[157] Raskin A., Lange S., Banares K., Lyon R.C., Zieseniss A., Lee L.K., Yamazaki K.G., Granzier H.L., Gregorio C.C., McCulloch A.D., Omens J.H., Sheikh F. (2012). A novel mechanism involving four-and-a-half LIM domain protein-1 and extracellular signal-regulated kinase-2 regulates titin phosphorylation and mechanics. J. Biol. Chem..

[158] Lun A.S., Chen J., Lange S. (2014). Probing muscle ankyrin-repeat protein (MARP) structure and function. Anat. Rec. (Hoboken).

[159] Witt S.H., Labeit D., Granzier H., Labeit S., Witt C.C. (2005). Dimerization of the cardiac ankyrin protein CARP: implications for MARP titin-based signaling. J. Muscle Res. Cell Motil..

[160] Moulik M., Vatta M., Witt S.H., Arola A.M., Murphy R.T., McKenna W.J., Boriek A.M., Oka K., Labeit S., Bowles N.E., Arimura T., Kimura A., Towbin J.A. (2009). ANKRD1, the gene encoding cardiac ankyrin repeat protein, is a novel dilated cardiomyopathy gene. J. Am. Coll. Cardiol..

[161] Miller M.K., Bang M.L., Witt C.C., Labeit D., Trombitas C., Watanabe K., Granzier H., McElhinny A.S., Gregorio C.C., Labeit S. (2003). The muscle ankyrin repeat proteins: CARP, ankrd2/Arpp and DARP as a family of titin filament-based stress response molecules. J. Mol. Biol..

[162] Bogomolovas J., Gasch A., Simkovic F., Rigden D.J., Labeit S., Mayans O. (2014). Titin kinase is an inactive pseudokinase scaffold that supports MuRF1 recruitment to the sarcomeric M-line. Open Biol..

[163] Lange S., Xiang F., Yakovenko A., Vihola A., Hackman P., Rostkova E., Kristensen J., Brandmeier B., Franzen G., Hedberg B., Gunnarsson L.G., Hughes S.M., Marchand S., Sejersen T., Richard I., Edstrom L., Ehler E., Udd B., Gautel M. (2005). The kinase domain of titin controls muscle gene expression and protein turnover. Science.

